# Enzymatic Defluorination of Perfluorooctanoic Acid by an Evolutionarily Distinct Haloacid Dehalogenase

**DOI:** 10.21203/rs.3.rs-10043589/v1

**Published:** 2026-07-22

**Authors:** OMOWUNMI Sadik

**Affiliations:** New Jersey Institute of Technology

## Abstract

Here, we demonstrate the successful purification of haloacid dehalogenase type II (HAD-II) enzyme, validating its catalytic capacity to directly mediate the cell-free defluorination of long-chain perfluorooctanoic acid (PFOA) by systematically cleaving the resilient C-F bond. While conventional remediation strategies rely on energy-intensive chemical methods, biological alternatives are limited to sluggish whole-microbiome consortia. We discovered a novel HAD-II enzyme from Achromobacter mucicolens harvested from PFAS-contaminated lacustrine sediment. Within 24 hours, the recombinant enzyme achieved cell-free PFOA defluorination, releasing 0.55 ppm of free fluoride (17% yield). Structural and phylogenetic analyses reveal that this HAD-II belongs to a deeply divergent lineage sharing only 25% sequence identity with the previously characterized Delftia homologue while preserving the core HAD-like catalytic fold. Comparative molecular docking elucidated that PFOA adopts a productive binding orientation near the conserved catalytic Asp15 within the A. mucicolens active-site pocket. Together, our work establishes a clean mechanistic paradigm for targeted environmental biotechnology.

## Introduction

Per- and polyfluoroalkyl substances (PFAS) represent one of the most pervasive anthropogenic environmental crises of the modern era. Due to the extreme thermodynamic stability of the carbon-fluorine (C-F) bond (~ 485 kJ/mol), these compounds exhibit exceptional resistance to degradations^1,2^. The environmental mobility of PFAS has led to its ubiquitous contamination across diverse environments, including remote areas^3,4^. These xenobiotics bioaccumulate in trophic webs due to prolonged biological half-lives and strong binding to circulating proteins^5,6^. PFAS encompasses a chemically diverse group of over 4,000 compounds, and non-polymeric PFAS like PFOA and PFOS^7,8^ remain dominant due to strong C-F bonds, serving as benchmark targets for remediation^9,10^.

To date, conventional non-enzymatic remediation strategies have proven insufficient to sustainably address the global burden explored to degrade PFAS^11–18^. Although approaches like electrochemical removal^15,19–21^, and adsorption^22,23^ primarily achieve partial removal or conversion of PFAS, these engineered interventions are severely constrained by prohibitive operational costs, immense energy penalties, and the accidental generation of shorter-chain, toxic fluoro-alkane byproducts. While whole-cell bacterial bioremediation offers a theoretically scalable revenue, its real-world execution is limited due to slow and incomplete biotransformation over extended multi-month timelines^24,25^. The underlying constraints are largely driven by restricted cellular uptake of PFAS, the metabolic toxicity of accumulated intracellular fluoride, and pressure on bacteria to develop fluoride resistance mechanisms rather than the synthesis of direct C-F cleaving pathways^26^.

Enzyme-based defluorination represents a sustainable alternative to conventional remediation techniques by employing naturally occurring biochemical reactions that operate under mild conditions without reliance on harsh chemicals or energetic inputs^25–30^. Peroxidases and laccases are among the most widely studied enzymes, exploiting their lignin-degrading capabilities to interact with the strong C-F bonds found in PFAS^31–35^. These enzymes tend to lose activity under working conditions, and their performance depends on external cofactors (H_2_O_2_ or mediators), where imbalances may lead to persistent by-product formation and insufficient defluorination performance or even enzyme inactivation^26,36^. Moreover, most reported studies are conducted under simplified laboratory conditions using spiked aqueous solutions rather than environmental samples, which fail to reflect the complexity of natural water contamination scenarios.

**Figure 1:**
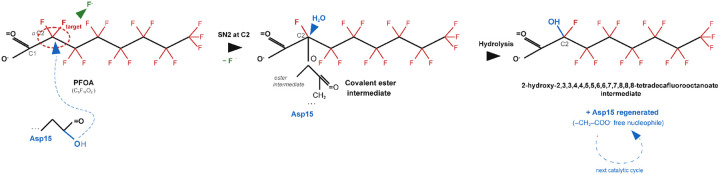
Catalytic mechanism of HAD-II dehalogenase-mediated defluorination of PFOA at the α-carbon. Red bonds and labels denote fluorine atoms and the target C2–F bond; blue denotes the Asp15 nucleophile, the attacking water molecule, and the newly introduced hydroxyl group; green denotes fluoride departure.

To overcome the vulnerabilities of unselective radical oxidation, attention must shift toward target-specific hydrolytic enzymes. Haloacid dehalogenases (HADs), as a major subclass of dehalogenase enzymes, are responsible for catalyzing the breakdown of C-F bonds in halogenated organic compounds (HOCs), whose environmental persistence is largely attributed to the strong stability of C-X bonds, especially the highly recalcitrant C-F bond^37–39^. HADs mediate the conversion of 2-haloacids to their corresponding hydroxy acids and exhibit broad halogenated substrate versatility, underscoring their potential in remediation processes^38,40,41^. HAD enzymes are characterized by a conserved Rossmann-like α/β-fold, essential catalytic residues, and a dynamic cap region that promotes efficient substrate interaction and drives their broad catalytic functionality and robustness^41,42^. While natural microbial dehalogenation processes face limitations in environmental settings, the structural flexibility and substrate versatility of HAD enzymes position them as attractive tools for transforming persistent fluorinated contaminants^43,44^. PFOA (C_8_F_15_O_2_^−^), a fully perfluorinated eight-carbon carboxylate, undergoes defluorination initiated at the α-carbon (C2), which bears two fluorine atoms. Mechanistically, the active-site nucleophile Aspartate (Asp) nucleophile performs a backside S_N_2 attack on C2, displacing one fluoride ion (F^−^) and forming a covalent ester intermediate between the enzyme and the substrate. Subsequently, a water molecule hydrolyzes the ester bond, releasing the first defluorination product, which is 2-hydroxy-2,3,3,4,4,5,5,6,6,7,7,8,8,8 tetradecafluorooctanoate intermediate, in which C2 now carries a hydroxyl group and one remaining fluorine, while regenerating the catalytic Asp residue for other defluorination reactions (Fig. 1)^42^. Recent studies identified PFAS-degrading bacteria from contaminated soils and linked them to dehalogenase genes through genome analysis, relying on bacterial culture without enzyme purification, leaving the biochemical reality of direct enzymatic PFAS defluorination unresolved^45^.

Here, we report the discovery, isolation, and structural characterization of a novel haloacid dehalogenase type II (HAD-II) enzyme harvested from a native *Achromobacter mucicolens* strain within a PFAS-contaminated lacustrine sediment in New Jersey. Moving beyond descriptive genomics, we successfully cloned, recombinantly expressed, and purified HAD-II to evaluate its cell-free PFOA defluorination performance. Using ion chromatography/ion-selective electrode, and mass spectrometry, we tracked the stepwise defluorination cascade of PFOA. Our structural analyses show that *A. mucicolens* variant occupies an evolutionarily distinct zone, sharing only 25% sequence identity with previously characterized *Delftia* dehalogenases while retaining a spacious hydrophobic pocket that accommodates long-chain PFAS, where all other known homologues suffer steric exclusion.

## Results

### Microbial community dynamics and discovery of a candidate HAD-II gene

**Figure 2:**
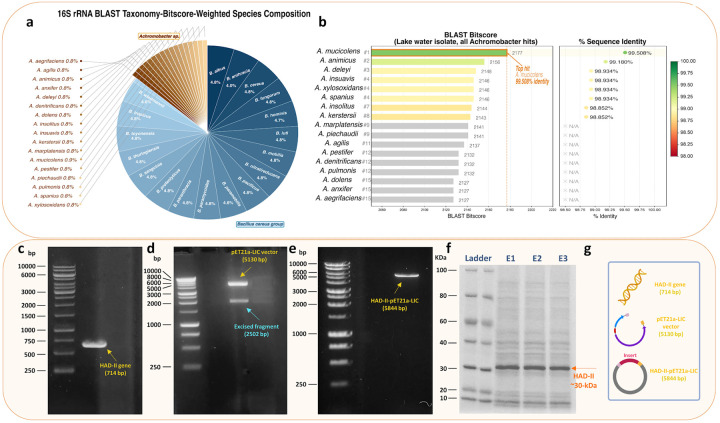
**(Top panels) Genus-level taxonomic composition of seven bacterial isolates by 16S rRNA gene sequencing**. **a)** The pie chart summarizes genus-level assignments for all seven isolates (n = 7). Six isolates (P_1_-P_6_; 85.7%) were assigned to the Bacillus cereus group and one isolate (S_C_; 14.3%) was assigned to *Achromobacter sp*. based on the highest-scoring BLASTN hit against the NCBI 16S rRNA RefSeq database. Annotation boxes list all candidate species recovered from the top-25 BLAST hits for each genus. For the *Bacillus cereus* group (P_1_-P_6_), 19 candidate species were identified with percentage sequence identities of 96.94–100.00%; species-level resolution within this group is not achievable by 16S rRNA alone. For the *Achromobacter* isolate (from the lake), 17 candidate species were identified with percentage sequence identities of 98.85–99.51%; the top hit was *A. mucicolens* (99.51%). **b)** 16S rRNA-based taxonomic identification of a bacterial isolate from the S_C_ sample. BLAST bitscore for all 17 *Achromobacter* candidate species recovered from the top-25 BLASTN hits against the NCBI 16S rRNA RefSeq database. Bars are colored by percentage sequence identity (RdYlGn scale, 98–100%); grey bars indicate hits for which alignment-block identity was not reported (BLAST ranks 11–25). The dashed vertical line marks the top-hit bitscore (2177). BLAST rank is shown to the left of each bar. **(Bottom panels) Cloning and expression of the HAD-II dehalogenase gene in a pET21a-LIC expression vector. c)** Agarose gel electrophoresis confirming PCR amplification of the HAD-II gene (714 bp) from the bacterial isolate template. **d)** Restriction digest of the pET21a-LIC vector (5,130 bp) showing the excised fragment (2,502 bp) following linearization, confirming successful vector preparation. **e)** Agarose gel confirming the final recombinant construct HAD-II-pET21a-LIC (5,844 bp) after ligation and transformation. **f)** SDS-PAGE analysis of recombinant HAD-II protein expression in *E. coli* BL21(DE3). Elution fractions E1, E2, and E3 show a prominent band at ~30 kDa, consistent with the predicted molecular weight of HAD-II (26.3 kDa untagged; ~29–30 kDa with His-tag). **g)** Schematic of the cloning strategy: the 714 bp HAD-II gene was inserted into the 5,130 bp pET21a-LIC vector to generate the 5,844 bp recombinant expression construct HAD-II-pET21a-LIC.

To evaluate the selective enrichment of microbial communities capable of growth on PFOA as a sole carbon source, full-length 16S rRNA gene clone libraries were constructed from enrichment cultures of six soil samples (P _1_ -P _6_) and one productive lake sample (S _C_). Three of the original four lake sediment samples (S _A_, S_B_, S_D_) failed to yield viable enrichment cultures under the experimental conditions and were excluded from further metagenomic processing. Clone library analysis of the S_C_ samples revealed distinct microbial community compositions between the soil-derived and lake-derived enrichments. In the soil enrichment cultures (P_1_-P_6_), the dominant sequences were affiliated with the genus Bacillus (phylum Firmicutes) (Fig. 2a), consistent with the nutrient-rich character of agricultural soils, which favor fast-growing, spore-forming generalists capable of surviving high chemical burdens. In contrast, the PFOA-contaminated lacustrine sediment matrix (S_C_) was dominated by sequences affiliated with the genus Achromobacter (class Betaproteobacteria) (Fig. 2b), implying that this genus is selectively enriched in the nutrient-limited lake environment, where PFOA is an important carbon source. This is consistent with reports of oligotrophic, pollutant-exposed aquatic environments being selective for specialized degraders over copiotrophic soil taxa^46^. The convergence of 16S rRNA community profiling in the same enrichment culture provides strong contextual evidence that Achromobacter mucicolens harbors a functional dehalogenase that may contribute to PFOA metabolism under carbon-limited conditions. To map this metabolic candidate, genome-based screening was conducted to detect enzymes with potential dehalogenase activity. A high-probability candidate gene encoding an L−2-haloacid dehalogenase type II (HAD-II) framework was successfully identified on a high-coverage contig taxonomically assigned to Achromobacter mucicolens. The protein encoded in this gene (WP_280147839.1; 238 amino acids) shares only 26.8% and 26.0% pairwise sequence identity with the previously characterized HAD-II dehalogenases from Delftia acidovorans (WP_011137954.1) and Xanthobacter dioxanivorans (WP_203197032.1), respectively, placing it in the twilight zone of sequence similarity. This structural insulation strongly suggests that the enzyme represents a unique branch of the HAD-II subfamily that has evolved a specialized catalytic architecture to destabilize the highly polarized C-F bonds of long-chain targets. To validate the functional reality of this candidate, we proceed to express the HAD-II dehalogenase gene, purify the respective protein, and perform cell-free biochemical evaluation.

### Cloning, recombinant expression, and verification of HAD-II

To express HAD-II, we cloned and purified the protein by amplifying the putative HAD-II gene fragment (~ 714 bp) by PCR (Fig. 2c). This was followed by double digestion of the pET21a-LIC plasmid with BspOI and SacI, which generated the expected linearized vector (~ 5130 bp) and excised fragment (~ 2502 bp), which supports successful vector preparation for ligation (Fig. 2d). Subsequently, ligation of the amplified HAD-II inserts into the digested pET21a-LIC vector resulted in a recombinant HAD-II-pET21a-LIC plasmid (~ 5844 bp), which was verified by agarose gel electrophoresis before sequence confirmation (Fig. 2e). The recombinant plasmid was then transformed into E. coli DH5α for plasmid amplification and further validated through DNA sequencing to confirm correct insertion and sequence integrity (Fig. S1).

To purify the protein, the verified recombinant HAD-II-pET21a-LIC construct was introduced into E. coli BL21(DE3) cells for inducible heterologous expression. Following IPTG-mediated expression of the recombinant protein in E. coli BL21(DE3), purification using a HisTrap^™^ HP affinity column enabled efficient recovery of His-tagged HAD-II enzymes. A prominent protein band at an apparent molecular weight of ~ 30 kDa was observed in Sodium dodecyl sulfate-polyacrylamide gel electrophoresis (SDS-PAGE) analysis, demonstrating successful expression and purification of HAD-II (Fig. 2f). The detected protein band closely matched the calculated molecular weight of recombinant HAD-II (26.3 kDa untagged; ~29–30 kDa with His-tag), which validates precise gene cloning, effective expression, and purification of the target dehalogenase enzyme. The recombinant expression strategy employing pET21a-LIC and E. coli BL21(DE3) enabled efficient production of soluble recombinant HAD-II protein for downstream functional and stability analyses.

### Quantitative profiling of whole-cell and cell-free defluorination activity

**Figure 3:**
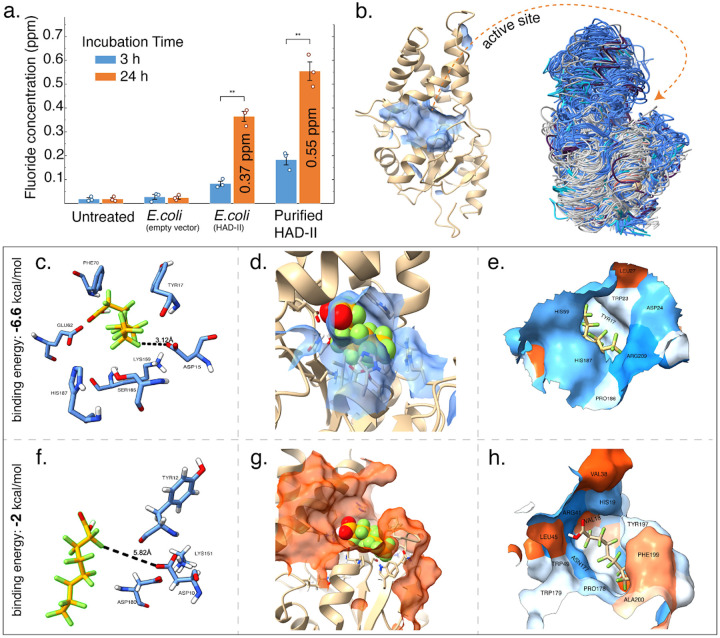
**(Top panels) Defluorination activity and structural basis of PFOA binding by the *A. mucicolens* HAD-II enzyme. a)** Fluoride ion release during bacterial culture and purified recombinant HAD-II defluorination assays following incubation with PFOA. Data represent the mean ± standard deviation of triplicate experiments calculated from combined ISE and IC measurements. **b)** Left: AlphaFold4-predicted three-dimensional structure of *A. mucicolens* HAD-II enzyme (pTM = 0.935, pLDDT = 0.931) rendered as a ribbon diagram with active-site cavity shown as a transparent blue surface. Right: Foldseek structural superposition of 50 top-scoring PDB homologues (TM-score > 0.70) onto the predicted HAD-II structure, colored from white to dark blue by increasing TM-score; the dashed orange arrow indicates the conserved active-site region. **(Bottom panels) Molecular docking of PFOA to HAD-II dehalogenase from *Achromobacter mucicolens* (HAD-II; WP_280147839.1) and reference HAD enzyme from *Delftia acidovorans* (WP_011137954.1). c-e)** Docking of PFOA to HAD-II. PFOA (green fluorine, orange carbon) binds within the active-site pocket and is positioned 3.12 Å from catalytic Asp15 within hydrogen-bonding distance. Key active-site residues include Tyr17, Phe70, Glu62, Lys159, Ser185, and His187. **d)**Surface representation of the protein with PFOA shown as spheres (green fluorine, red oxygens, orange carbons), docked within a well-defined hydrophilic cavity (blue surface). **e)** Electrostatic surface map of the binding pocket showing PFOA accommodated in a predominantly electropositive (blue) cavity, with Tyr17, Trp23, Asp24, His59, His187, Pro186, Arg209, and Leu27 contributing to pocket architecture. The AutoDock Vina binding affinity score is −6.679 kcal/mol. **f-h)** Docking of PFOA to WP_011137954.1 (*Delftia acidovorans*). **f)** PFOA displaced from the catalytic site; the closest catalytic residue, Asp10, is 5.82 Å from the ligand, too distant for nucleophilic attack. The PFOA pose is oriented toward a peripheral region near Asp180, Tyr12, and Lys151. **g)** Surface representation showing PFOA docked to a shallow, electronegative (orange) surface region outside the canonical active-site cavity. **h)** Electrostatic surface map confirming PFOA binding to a hydrophobic peripheral pocket, surrounded by Val38, His19, Arg41, Val18, Leu45, Tyr197, Trp49, Asn177, Pro178, Trp179, Phe199, Ala200, and Leu45, none of which correspond to the catalytic machinery (The binding affinity score is −2.0 kcal/mol).

After successful production of soluble recombinant HAD-II enzyme, we proceeded to test its putative defluorination activity using parallel whole-cell and purified enzyme systems (Fig. 3a). Fluoride ion release was determined in triplicate (n = 3, mean ± SD) using both Ion-Selective Electrode (ISE) potentiometry and Ion Chromatography platforms. Only minimal fluoride ions were detected in the untreated water and treated water with empty-vector E. coli BL21(DE3) samples, exhibiting fluoride concentrations below ~ 0.05 ppm during the incubation period. These findings confirm negligible background fluoride interference from culture medium and host strain under the tested conditions. In contrast, recombinant E. coli expressing HAD-II demonstrated a clear increase in fluoride ion release in response to PFOA treatment from ~ 0.08 ppm at 3 h to ~ 0.37 ppm at 24 h of incubation, which demonstrates time-dependent whole-cell defluorination activity associated with HAD-II expression. The significant increase in fluoride ion release in the HAD-II-expressing sample relative to the empty-vector control suggests functional PFAS defluorination activity of the HAD-II enzyme.

When evaluated under purified, cell-free conditions, recombinant HAD-II demonstrated enhanced defluorination activity, generating approximately 0.18 ppm fluoride after 3 h, which increased to ~ 0.56 ppm following 24 h incubation (17% yield from a 5 ppm PFOA sample). The increased fluoride generation detected in the purified enzyme assay likely suggests that removal of cellular barriers enhanced substrate availability and enzymatic accessibility to PFOA (Fig. 3a). Collectively, the time-dependent increase in fluoride ion concentration supports the HAD-II capability to destabilize and cleave the C-F bond of long-chain perfluorinated carboxylates. Also, low fluoride concentrations in the negative control samples support the enzymatic origin of fluoride release observed in the HAD-II reaction systems. Similarly, the cell-free media control showed no change in fluoride concentration, ruling out spontaneous chemical defluorination and physical adsorption. Thus, our results confirm our initial hypothesis that HAD-II is responsible for clear time-dependent defluorination of PFOA. We now proceed to investigate the spatial configuration and binding dynamics of PFOA within the HAD-II active site pocket.

### Comparative docking and functional analysis of PFAS-associated HAD enzymes

The PFAS defluorination literature contains no report of an Achromobacter strain degrading PFOA, PFOS, or any PFAS. The organisms reported include Acidimicrobium sp. A6^47^, pure Pseudomonas strains^48^, Labrys portucalensis^49^, Delftia acidovorans^50^, but not Achromobacter. This study provides the first evidence of an Achromobacter enzyme capable of PFOA binding and defluorination, extending the known degradative capabilities of this genus beyond conventional pollutants^51^. Harris et al. reported Delftia HAD activity on PFOA; an observable amount of fluoride release was observed, but no kinetics or structure were analyzed^45^. Delftia acidovorans literature warrants particular attention as it directly informs the comparative analysis presented here. Harris et al. first reported that D. acidovorans D4B, isolated from PFAS-contaminated soil. However, subsequent biochemical characterization by Farajollahi et al. of five recombinantly expressed D4B dehalogenases (DeHa1−5) found that none defluorinated PFOA even after seven days of incubation^51^. Only DeHa2 (a haloacid dehalogenase) and DeHa4 (a fluoroacetate dehalogenase) showed defluorination activity, and only toward short-chain, partially fluorinated substrates, which are monofluoroacetate and difluoroacetate. Docking PFOA to the DeHa2 AlphaFold2 model placed the ligand outside the active site (−3.8 kcal/mol, non-productive), and PFOA binding to DeHa4 was thermodynamically unfavorable (+ 13.1 kcal/mol). These findings are consistent with our own docking results for Delftia HAD (WP_011137954.1), in which PFOA failed to approach the catalytic Asp10 of the Delftia HAD enzyme (5.82 Å distance; binding affinity−2.0 kcal/mol). Together, the biochemical and computational evidence indicate that the Delftia HAD enzymes characterized to date lack the active-site geometry required for productive PFOA engagement. The enzyme reported here, HAD-II (WP_280147839.1) from Achromobacter mucicolens, docks PFOA within 3.12 Å of the catalytic Asp15 with a binding affinity of−6.679 kcal/mol (Fig. 3c, d, and e), suggesting a qualitatively different substrate-binding capacity despite sharing only 26.8% sequence identity with the Delftia counterpart. The study enzyme HAD-II and its Delftia acidovorans homologue have low pairwise sequence identity (26.8%). Three-dimensional structures for both proteins were predicted using AlphaFold4 and performed structural homology searches against the RCSB Protein Data Bank (PDB) using Foldseek. Both predicted models were of high quality, with predicted TM-scores (pTM) of 0.935 and 0.951 and mean pLDDT values of 0.931 and 0.956 for HAD-II (A. mucicolens) and Delftia HAD, respectively, indicating reliable structural predictions (Fig. S2). Our docking results provide a comparison to Delftia HAD, which displays why the Delftia enzyme may have limited activity due to being off the catalytic site (Asp) in Fig. 3f, g, and h.

### Conformational and Thermal Stability Analysis of HAD-II

**Figure 4:**
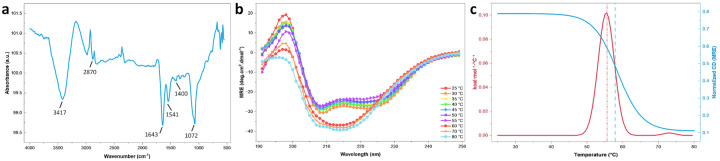
Structural and thermal characterization of recombinant HAD-II enzyme. **a)** FTIR spectrum of purified HAD-II exhibiting characteristic protein-associated absorption bands. **b)** Far-UV CD spectra of HAD-II, depicting temperature-dependent conformational changes. **c)** DSC thermogram and normalized CD thermal transition profile, revealing melting temperature.

We next initiated a comprehensive biophysical characterization to probe the thermal and conformational stability profiles of HAD-II under varying environmental conditions. The characteristic absorption bands of proteins are displayed by the FTIR spectrum of purified HAD-II enzyme (Fig. 4a). The Amide I band at ~ 1643 cm^−1^ with strong dependency on secondary structure arises from the C = O stretching vibration of the peptide backbone. The position of this band indicates a mixed secondary structure (α-helix, β-sheet, and random coil), which is consistent with typical enzyme conformations. Specifically, this reflects the conserved Rossmannoid fold of the HAD superfamily, which contains a central core of parallel β-strands surrounded by an α-helix. The Amide II band at ~ 1540 cm^−1^, which corresponds to N-H bending and C-N stretching vibrations, confirms the protein backbone presence. Additional bands at ~ 2870 cm^−1^ and ~ 1400 cm^−1^ are associated with C-H stretching and bending modes of aliphatic side chains, respectively. The peak at ~ 1027 cm^−1^ corresponds to C-O stretching vibrations, which may originate from amino acid side chains. The presence of water in the sample results in a broad band at ~ 3417 cm^−1^ originating from O-H stretching vibrations. Overall, the FTIR findings suggest that the protein maintains its structural integrity, with characteristic functional groups and a secondary structure consistent with a structured protein (Fig. 4a).

The temperature-dependent secondary structural stability of HAD-II was assessed by implementing Far-UV CD spectroscopy (Fig. 4b). The CD spectra displayed the characteristic double minima near 208 and 222 nm at temperatures 25–45°C, indicating a secondary structure enriched in α-helical content^52^. As the temperature increases, the observed changes in the ellipticity profile suggest partial unfolding and conformational changes within the HAD-II structure upon thermal exposure. Specifically, the CD spectra show a transition from a predominantly α-helical signature to a spectrum dominated by a single minimum near 215 nm. This suggests that the partial unfolding of outer α-helices at higher temperatures, while the internal β-sheet core of the Rossmannoid fold exhibits higher thermal stability, maintaining its structural signature up to 80°C^52^. This gradual conformational transition suggests that the HAD-II enzyme retains partial structural integrity over the evaluated temperature range. Using the CD spectra (Fig. 4b), normalized mean residue ellipticity (MRE) values at 222 nm were applied to generate the thermal denaturation profile (Fig. 4c), which revealed a melting temperature (T_m_) of 57.9017°C for the HAD-II enzyme.

The thermal stability of HAD-II was also evaluated by DSC analysis (Fig. 4c). A distinct endothermic transition peak (at approximately 55.6°C) was observed in the DSC spectrogram, which corresponds to the thermal unfolding of the HAD-II enzyme. The observed denaturation temperature measured by DSC was in close agreement with the T_m_ value determined from CD-based unfolding analysis (~ 57.9°C), highlighting a close correlation between the two biophysical approaches and further validating the observed thermal unfolding behavior of the HAD-II protein (Fig. 4c).

**Figure 5:**
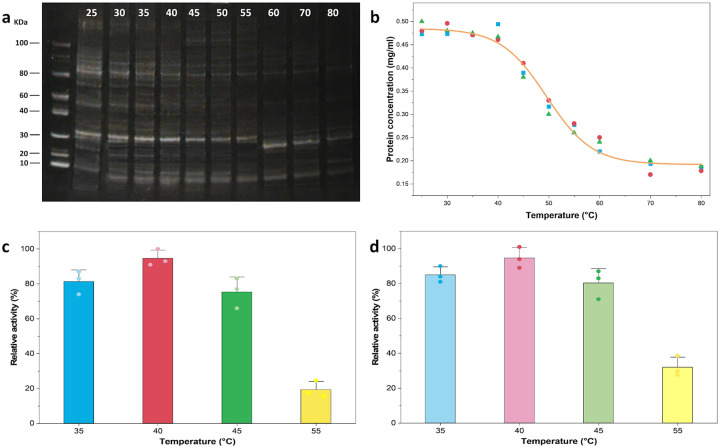
Thermal durability and defluorination efficiency analysis of HAD-II enzyme. **a)** CETSA-based SDS-PAGE analysis of HAD-II upon incubation at increasing temperatures. **b)** BCA-based quantification of HAD-II protein concentration following CESTA-based thermal treatment, **c)** Temperature-dependent catalytic efficiency profile of HAD-II normalized relative to the 40 °C condition. **d)** Temperature-dependent thermal stability analysis of HAD-II following pre-incubation prior to activity measurement. Data represents the mean ± standard deviation of triplicate experiments.

A CETSA-based thermal aggregation analysis combined with SDS-PAGE visualization was performed to assess the temperature-dependent stability of HAD-II (Fig. 5a, b). Increasing incubation temperature resulted in a gradual decrease in soluble HAD-II band intensity, reflecting progressive thermal denaturation and aggregation of the enzyme. Within the 25–55°C range, the HAD-II exhibited minimal loss of soluble band intensity, while maintaining a consistent migration pattern (~ 30 kDa), suggesting that the overall molecular mass of the soluble protein fraction is preserved. Consistently, Fig. 5b demonstrated only minor changes in soluble protein concentration measured by the BCA assay up to ~ 40°C, whereas a gradual decrease becomes apparent at higher temperatures, particularly above ~ 45–50°C. As insoluble aggregates were removed during post-heating centrifugation, the reduction in both band intensity and soluble protein concentration primarily reflects thermal aggregation of the HAD-II enzyme rather than direct protein degradation (Fig. 5a, b). However, exposure to temperatures above ~ 55°C resulted in the appearance of weaker lower-molecular-weight bands (< 30 kDa), indicating thermal destabilization and irreversible protein unfolding (Fig. 5a). The aggregation midpoint identified by CETSA and BSA analyses was detected within the ~ 55–60°C range, which corresponded well with the melting temperatures determined through CD and DSC analyses. This close agreement supports the moderate thermal stability of HAD-II and suggests preservation of conformational stability over a relatively broad temperature range. Overall, HAD-II maintains substantial structural stability under moderate thermal conditions while exhibiting progressive loss of solubility at temperatures above 55°C.

### Evaluation of HAD-II Biochemical Behavior

We next tracked the biochemical behavior of HAD-II based on the previously obtained thermal stability profile from CETSA analysis (Fig. 5a, b). The effects of temperature on HAD-II catalytic performance and thermal stability were evaluated within the 35–55°C range (Fig. 5c, d). The defluorination activity obtained at 40°C after 24 h incubation was used as a reference condition to calculate relative enzymatic activities for Fig. 5c and d. Regarding temperature-dependent activity analysis (Fig. 5c), recombinant HAD-II exhibited the highest relative activity at 35°C (~ 85%), while enzymatic activity reached ~ 75% at 45°C. Incubation at 55°C resulted in a notable reduction in enzymatic activity, where catalytic performance decreased to approximately 20%. The observed activity profile indicates HAD-II retains catalytic efficiency across moderate temperatures, while the pronounced activity loss near 55°C is consistent with the melting temperatures (~ 56–57°C) determined by CD and DSC analyses (Fig. 4b, c). This reduction is also supported by the CETSA/BCA results (Fig. 5a, b), where the measurable soluble protein fraction decreased due to elevated temperature, which suggests that partial unfolding and aggregation around 55°C reduce the availability of catalytically active HAD-II for PFOA defluorination.

Thermal stability analysis following 4 h pre-incubation at different temperatures before activity measurement (Fig. 5d) demonstrated a similar trend to the temperature-dependent activity assay (Fig. 5c). HAD-II remained at approximately 85% relative activity following pre-incubation at both 35 and 45°C, which suggests favorable thermal tolerance within this moderate temperature range. However, pre-incubation at 55°C led to a decline in activity to approximately 35%, reflecting notable thermal inactivation.

**Figure 6:**
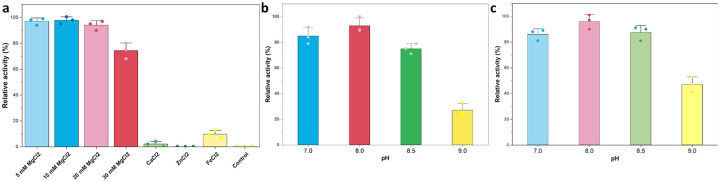
Biochemical characterization of recombinant HAD-II. **a)** Effect of divalent cations and MgCl_2_ concentration on HAD-II enzymatic activity normalized relative to the 5 mM MgCl_2_ condition. **b)** pH-dependent catalytic efficiency profile of HAD-II normalized relative to the pH 8.0 condition. **c)** pH stability profile of HAD-II following pre-incubation at different pH conditions before activity measurement. Data represent the mean ± standard deviation of triplicate experiments.

To profile the cofactor dependency of HAD-II, we screened its catalytic activity across a 5–30 mM concentration gradient of MgCl_2_, along with several alternative metal cofactors (Fig. 6a). Relative activities were normalized based on the reaction containing 5 mM MgCl_2_, defined as 100% activity. HAD-II displayed maximal relative activity in the presence of 10 mM MgCl_2_ (relative activities close to 100%), while increasing MgCl_2_ concentration above 10 mM resulted in a gradual decline (with ~ 75% relative activity at 30 mM MgCl_2_), which indicates the partial inhibition of catalytic efficiency due to excessive MgCl_2_ concentration. Among the alternative tested divalent cations, Ca^2+^ and Fe^2+^ supported only minimal enzymatic activity, while Zn^2+^ resulted in nearly complete loss of catalytic function. The negligible activity in the EDTA-treated control demonstrates the requirement of divalent metal ions for HAD-II function.

The influence of pH on HAD-II enzymatic activity and stability was studied within the pH 7.0–9.0 range using two HEPES and Tris–HCl buffer systems (Fig. 6b, c). The reaction at pH 8.0 was used as a reference to calculate relative activities at other pH values. The observed maximal catalytic activity at pH 8.0 indicated the favorability of mildly alkaline conditions for enzymatic defluorination. For pH-dependent activity analysis (Fig. 6b), while HAD-II retained ~ 85% relative activity at pH 7.0 and ~ 75% activity at pH 8.5, a pronounced reduction in enzymatic performance was observed under strongly basic environments at pH 9.0. These findings reveal the negative effect of extreme alkaline conditions on HAD-II catalytic efficiency. A similar activity pattern was observed during pH stability analysis following 4 h pre-incubation under different pH conditions before activity measurement at pH 8.0 (Fig. 6c). Between pH 7.0 and 8.5, the HAD-II enzyme exhibited approximately 85% activity relative to the pH 8.0 reference condition. Enzyme exposure to pH 9.0 resulted in a marked reduction (around 55%) in relative activity, suggesting reduced structural stability under highly alkaline conditions. The results indicate that HAD-II preserves substantial catalytic activity and conformational stability across near-neutral to mildly alkaline environments, with optimal enzymatic performance near pH 8.0. The loss of catalytic efficiency and stability at pH 9.0 indicates that elevated alkaline environments may disrupt active-site interactions and conformational integrity, which is required for effective PFAS defluorination.

### Screening of PFOA defluorination products by MS analysis

The untreated PFOA control was analyzed by direct-infusion negative-ion MS to establish the background profile prior to enzymatic defluorination. A dominant [PFOA-H] ^−^ ion at m/z 412.9 was observed in Fig. S3a, along with characteristic perfluoroalkyl fragment ions at m/z 368.9, 268.9, 218.9, 168.9, and 118.9, which arise from in-source dissociation of PFOA through decarboxylation and sequential cleavage of perfluoroalkyl units. This baseline spectrum confirmed the expected PFOA ionization pattern and served as the baseline to evaluate spectral changes after enzymatic defluorination^15^. In parallel, the MS spectrum of the enzymatically defluorinated PFOA sample is provided in Fig. S3b, showing new product-related ions that appeared in the treated sample with varying intensities (Table S1). Among observed products, while C_x_F_y_C(= O)COOH, and C_x_F_y_COOH are converted to deprotonated anions by proton loss, C_x_F_y_COF can undergo electron attachment to form radical anions. The PFOA defluorination pathway was suggested based on negative ESI-MS analysis. The PFOA parent anion at m/z 412.9 (C_7_F_15_COO^−^) undergoes enzymatic S_N_2 attack at the α-carbon/C_2_ position, as illustrated in Fig. 1. In this defluorination step, a fluorine atom is replaced with a hydroxyl group, forming an unstable 2-hydroxy-tetradecafluorooctanoate intermediate (CF_3_(CF_2_)_5_CF(OH)COO^−^), which may undergo rapid fluoride/HF elimination to generate an electrophilic α-keto intermediate, detected at m/z 390.9 (CF_3_(CF_2_)_5_C(= O)COO^−^). The proposed transformation is consistent with Fig. 1 and previously reported transformation pathways of fluorinated compounds^39,42,53^. Following formation of the α-keto intermediate, decarboxylation may generate the reactive perfluoroaldehyde CF_3_(CF_2_)_5_CHO, which is expected to hydrate in aqueous buffer to the transient CF_3_(CF_2_)_5_CH(OH)_2,_ due to the strong electron-withdrawing effect of the perfluoroalkyl chain. The subsequent oxidation of this hydrated species, mediated by dissolved oxygen or trace oxidizing species in the reaction medium, may then produce perfluoroheptanoate (PFHpA), detected at m/z 362.9 (C_6_F_13_COO^−^), with an in-source decarboxylation fragment at m/z 318.9 (C_6_F_13_^−^). PFHpA then re-enters the transformation cycle via sequential α-hydroxylation and HF elimination, undergoing a subsequent decarboxylation-oxidation to form perfluorohexanoate (PFHxA), detected at m/z 312.9 (C_5_F_11_COO^−^). The continued hydrolysis and HF-elimination steps suggest continuous shortening of the perfluoroalkyl chain, supported by detection of a downstream perfluoroacyl fluoride ion at m/z 215.9 (C_3_F_7_COF^−^)^15^. The observed ion series supports a continuing chain-shortening cycle of PFOA, while the absence of smaller terminal products may reflect the detection limits of the direct-infusion ion-trap MS method.

### Phylogenetic and sequence conservation analysis of the HAD-II Dehalogenase

**Figure 7:**
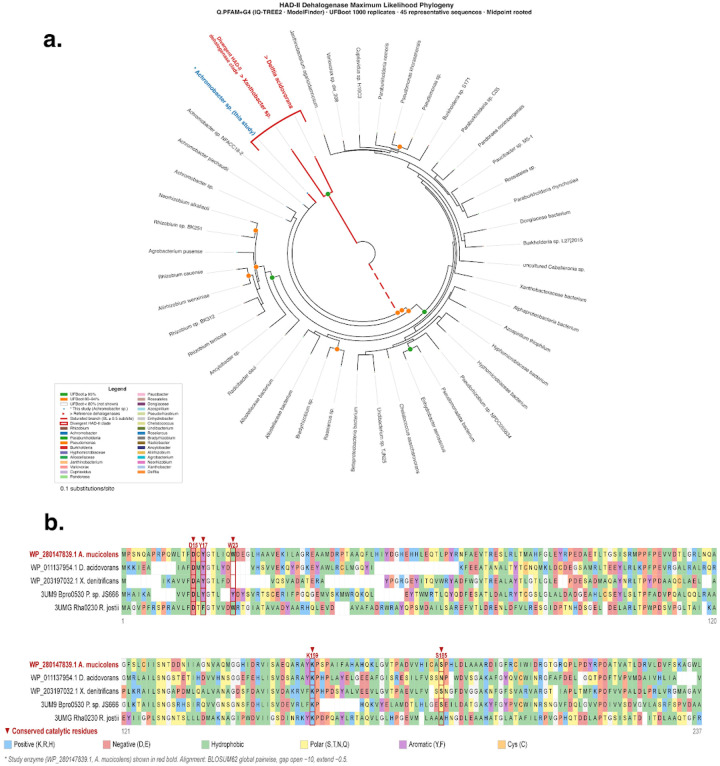
Maximum likelihood phylogeny of HAD family II dehalogenases. **a)** Circular phylogenetic tree of 45 representative HAD-II dehalogenase sequences inferred by IQ-TREE2 (v3.1.1) under the Q. PFAM+G4 substitution model selected by ModelFinder (BIC criterion). The tree is midpoint rooted. Branch support was assessed by ultrafast bootstrap (UFBoot; 1,000 replicates): filled green circles indicate UFBoot ≥ 95% and orange circles indicate UFBoot 80–94%; nodes with UFBoot < 80% are not marked. The dehalogenase characterized in this study (*Achromobacter sp.*;) and the two previously reported reference dehalogenases; *Delftia acidovorans* (WP_011137954.1) and *Xanthobacter sp*. (WP_203197032.1); are indicated by filled arrowheads (>). These three sequences form a distinct, deeply divergent clade (red bracket) that is separated from the main ingroup by a saturated branch (branch length ≥ 9.5 substitutions/site across all models tested; shown as a dashed red line, displayed capped at 1.0 for visual clarity). Tip labels are colored by genus. Scale bar represents 0.1 amino acid substitutions per site. Multiple sequence alignment of haloacid dehalogenase type II (HAD-II) homologs. **b) Multiple sequence alignment of WP_280147839.1 with four HAD-II dehalogenase homologues**. Sequences shown are: *Achromobacter mucicolens* WP_280147839.1 (this study, red bold), *Delftia acidovorans* WP_011137954.1, *Xanthobacter denitrificans* WP_203197032.1, Bpro0530 from *Polaromonas sp. JS666* (PDB: 3UM9), and Rha0230 from *Rhodococcus jostiiRHA1*(PDB: 3UMG). Alignment was generated using BLOSUM62 global pairwise alignment. Residues are colored by physicochemical class. Red boxes and triangles (▼) indicate conserved catalytic residues: Asp15 (nucleophile), Tyr17, Trp23, Lys159 (nucleophile stabilizer), and Ser185 (substrate carboxylate binding), numbered according to WP_280147839.1. Despite low overall pairwise sequence identity (18–27%), the catalytic framework is structurally and functionally conserved across all five sequences.

To trace the evolutionary origin of this unique biochemical profile and understand how such a highly active enzyme emerged from a sequence-divergent background, we next performed a comprehensive phylogenetic and sequence conservation analysis. The Achromobacter sp. dehalogenase characterized here does not cluster within the main Achromobacter ingroup but instead forms a distinct, deeply divergent clade together with the two previously characterized pollutant-degrading dehalogenases from Delftia acidovorans and Xanthobacter sp. The branch separating this clade from the remaining sequences is saturated (branch length ≥ 9.5 substitutions/site), indicating extreme evolutionary divergence (Fig. 7a). The moderate UFBoot support for the Achromobacter-Delftia sister relationship (64–87% across models) reflects genuine phylogenetic uncertainty at this deep node rather than a methodological artifact, as the topology is fully consistent across four independent substitution models. Sequence conservation analysis of 58 homologs revealed highly conserved motifs at positions 12–18 and 179–188. Specifically, residues D13 and D184 are strictly conserved, suggesting their critical role in the catalytic mechanism of this HAD-II dehalogenase, while variability in positions 30–43 indicates non-essential surface-exposed loops. Delftia and Xanthobacter share only ~ 27% identity with the newly discovered HAD-II enzyme, and this is clearly visible in the Multiple Sequence Alignment (MSA, Fig. 7b). Despite this, the conserved core positions are shared even with these distant relatives, confirming they are under strong purifying selection and likely essential for the HAD-II fold and catalytic mechanism.

## Summary

In this study, we identified and characterized a novel HAD-II family dehalogenase (WP_280147839.1) from Achromobacter mucicolens, a bacterium isolated from a PFAS-contaminated lake sediment. Structural, evolutionary, and computational data collectively demonstrate that the A. mucicolens dehalogenase possesses the essential HAD-II fold, highly conserved active-site residues (Asp15 and Tyr17), and a compatible binding geometry required for PFOA defluorination. Phylogenetic analysis of 101 HAD-II sequences placed the enzyme in a distinct clade, sharing only 26.8% and 26.0% pairwise sequence identity with the closest biochemically characterized defluorinases yet retaining the conserved catalytic triad (Asp15 nucleophile, Lys159, Tyr17) and halide-binding pocket (Arg·Asn·Trp/Phe) identified by ConSurf analysis as the most evolutionarily constrained positions in the alignment. Structural modelling with Boltz−2 (pTM = 0.935, pLDDT = 0.931) and Foldseek comparison against the PDB confirmed unambiguous adoption of the HAD-II fold (all 50 hits TM > 0.70), with a pairwise TM-score of 0.899 against the Delftia acidovorans homologue at only 26.8% sequence identity, demonstrating that structural conservation far exceeds sequence conservation across this enzyme family. Critically, the recombinant enzyme expressed in E. coli BL21(DE3) demonstrated measurable PFOA defluorination activity both in bacterial culture and cell-free systems. Bacterial cultures expressing HAD-II released significantly more fluoride ions than empty-vector controls, and purified recombinant protein generated approximately 0.18 ppm fluoride after 3 h, rising to ~ 0.56 ppm after 24 h incubation with PFOA under cell-free conditions, a time-dependent accumulation consistent with iterative enzymatic C-F bond cleavage at the α-carbon. Together, the convergence of sequence-independent structural homology to known defluorinases, conservation of the compact active-site geometry, and direct bench-top demonstration of fluoride release from PFOA establish HAD-II (WP_280147839.1) as a bona fide PFOA-defluorinating enzyme and A. mucicolens as a promising chassis for future bioremediation strategies targeting perfluoroalkyl acid contamination.

## Methods

### Reagents, strains, and cultivation conditions

All tested PFOAs were purchased from Sigma-Aldrich (St. Louis, MO, USA). 16S rRNA sequencing was performed by GENEWIZ (from Azenta Life Sciences). *E. coli* DH5α was commonly used as a host strain for molecular cloning, and strain BL21(DE3) was employed for functional protein overexpression. *Escherichia coli* (*E. coli*) competent cells (BL21/DE3) were obtained from Thermo Scientific. The cloning plasmid pET21a-LIC (Addgene #62303) was used as the cloning and expression vector, respectively. *E. coli* cells were usually cultured in Luria-Bertani (LB) medium (Sigma) supplemented with 100 μg/mL ampicillin, which was used for selection, and grown at 37°C and 200 rpm for 16 h.

#### Soil sampling and Lacustrine sediment sampling for microbial enrichment

Soil samples were collected from multiple environmental locations in New Jersey, including four distinct sampling points (S_A_, S_B_, S_C_, S_D_) within Pine Lake Park (**Fig. S4**), where PFAS-contaminated lake-associated soils were collected, as well as six field-contaminated soil samples (P_1_-P_6_) from defense-related facilities, to capture spatial variability. The selected sampling sites represented environmentally impacted areas associated with potential PFAS exposure, which originates from urban runoff, municipal wastewater, and other human-derived environmental sources frequently reported in the northeastern United States. Samples were homogenized and processed for microbial enrichment in PFAS-containing media. In brief, 1 g of each sample was resuspended in 5 mL of sterile 1/10 tryptic soy broth supplemented with 100 ppm PFOA, followed by vortexing to achieve uniform suspension and facilitate sedimentation of heavier soil particles. Aliquots of the soil suspensions were subsequently introduced into fresh PFAS-containing sterile medium and incubated under shaking conditions to facilitate selective microbial enrichment. Following settling, aliquots were transferred into microplate wells and subjected to a subsequent tenfold dilution in fresh medium to minimize biomass variability and enhance measurement sensitivity. Cultures were incubated at 30°C for up to 2 weeks, and optical density was monitored at 600 nm using a plate reader. For each of the ten sampling locations, four biological replicates were prepared, and the highest-performing cultures were selected in PFAS-supplemented medium. For molecular identification, E.Z.N.A. Soil DNA Kit (Omega Biotek, USA) was used for the extraction of total bacterial genomic DNA from the selected enriched cultures. Following this, DNA concentration and purity were assessed using a NanoDrop spectrophotometer (ng/μL). The samples were subsequently subjected to 16S rRNA gene sequencing to identify the enriched microbial populations.

##### 16S rRNA gene sequencing and taxonomic identification

Bacterial isolates were identified by Sanger sequencing of the 16S rRNA gene by GENEWIZ Azenta (NJ, USA). PCR amplification was performed using universal 16S rRNA primers, and the resulting amplicons were sequenced by Sanger sequencing. Sequence quality was assessed, and sequences were trimmed to remove low-quality terminal regions before database searching. Taxonomic identification was performed by querying each trimmed 16S rRNA sequence against the NCBI 16S rRNA RefSeq database using the Basic Local Alignment Search Tool (BLASTN; NCBI BLAST+ v2.x). Search parameters were set to retrieve the top 25 hits per query (max_target_seqs = 25), with default word size, gap penalties, and scoring matrix (match/mismatch: +1/−2). For each hit, the bitscore, percentage sequence identity, query coverage, and E-value were recorded. Genus-level assignment was based on the top-scoring BLAST hit. Six isolates (P_1_-P_6_) were assigned to the Bacillus cereus group (percentage identity: 96.94–100.00%; bitscore: 1874–2253) and one isolate (from the lake sediment) was assigned to *Achromobacter sp*. (top hit: *A. mucicolens*; percentage identity: 99.51%; bitscore: 2177). All top 25 BLAST hits for each isolate were retained for downstream analysis to capture the full range of candidate species. Bitscore-normalized species weights were computed for each isolate by dividing the bitscore of each hit by the sum of all 25 bitscores for that isolate, providing a relative confidence measure across candidate species. Taxonomic lineages for all 36 candidate species (19 *Bacillus*, 17 *Achromobacter*) were retrieved programmatically from the NCBI Taxonomy database using the Biopython Entrez interface (Biopython v1.x; NCBI Taxonomy, accessed May 2026).

##### Cloning of the HAD-II gene from Achromobacter mucicolens

Following microbial identification through 16S rRNA sequencing, genome-based screening was conducted to detect enzymes with potential dehalogenase activity. A putative haloacid dehalogenase type II (HAD-II) gene (714 bp) from *Achromobacter mucicolens* was identified, and subsequently synthesized (Integrated DNA Technologies, USA). PCR amplification of the synthesized construct was carried out using Phusion^™^ Plus DNA Polymerase in an Eppendorf MasterCycler (5333 model). The full-length coding sequence HAD-II was amplified using primers HAD-II-Forward (5′-/5Phos/TAAGCAGCTAGCATGCCTTCTAACCAAGCT-3′) and HAD-II-Reverse (5′-/5Phos/TGCTTAGAGCTCTCAGAGCCAGCCGGCTTTA-3′). The PCR-amplified HAD-II fragment and the pET21a-LIC vector were digested using Thermo Scientific FastDigest BspOI and SacI restriction enzymes to generate compatible sticky ends, followed by ligation of the insert into the linearized vector. The final recombinant plasmid, HAD-II-pET21a-LIC, was introduced into *E. coli* DH5α for plasmid amplification, and correct insertion was verified via DNA sequencing. The verified construct was then transferred into *E. coli* BL21(DE3) and selected on LB agar plates supplemented with ampicillin.

#### Recombinant production and purification of HAD-II enzyme

The LB medium supplemented with 50 mg/mL ampicillin was prepared for the cultivation of *E. coli* BL21(DE3) cells carrying the HAD-II-pET21a-LIC construct with shaking at 37°C until reaching an OD600 of 0.4–0.8. Then, the protein expression was induced using 1 mM IPTG, followed by incubation at 18°C under agitation for 48 h to facilitate production of His-tagged HAD-II. Bacterial cells were pelleted by centrifugation at 6,000 ×g for 30 min at 4°C, followed by removal of the supernatant. The harvested cell pellets were suspended in lysis buffer (50 mM Tris-HCl, 500 mM NaCl, 20 mM imidazole) enriched with lysozyme, DNase, RNase, and protease inhibitors, and mixed thoroughly to ensure complete homogenization. Sonication was performed in an ice-water bath to ensure efficient cell disruption while maintaining protein stability. The crude lysate was subjected to centrifugation at 15,000 ×g for 30 min at 4°C to pellet insoluble debris, allowing collection of supernatants containing the soluble protein fraction, which was further clarified by filtration through a 0.2 μm membrane, resulting in a cleared soluble lysate. The soluble fraction was introduced into a 5 mL HisTrap HP nickel affinity column (Cytiva) which was pre-equilibrated with binding buffer (50 mM Tris-HCl, 500 mM NaCl, 20 mM imidazole). The affinity resin was washed using 8 column volumes of wash buffer (50 mM Tris-HCl, 100 mM NaCl, 40 mM imidazole) and subsequently eluted using elution buffer containing 200 mM imidazole over 5 column volumes.

#### Biophysical and structural characterization of HAD-II Enzyme

##### Structural characterization by FTIR Spectroscopy

FTIR analysis of the purified HAD-II enzyme was performed using a Fourier Transform Infrared Spectrometer, IR Affinity-1 (Shimadzu, Japan). The purified enzyme solution was analyzed at room temperature, and spectra were recorded over the range of 400–5000 cm^−1^ with 64 scans at a resolution of 4 cm^−1^. Spectra were background-subtracted before analysis.

##### Conformational analysis using circular dichroism (CD) spectroscopy

CD measurements were performed on purified HAD-II enzyme using a JASCO J-1500 CD spectrophotometer (JASCO, Tokyo, Japan) with a Peltier temperature-control system. The far-UV CD profile of HAD-II was recorded between 190 and 250 nm in a 1 mm quartz cuvette under the following parameters: 1 nm bandwidth and a scanning speed of 100 nm·min^−1^. The enzyme was subjected to temperature-dependent CD analysis from 20 to 80°C. The HAD-II sample was equilibrated for 10 min at each selected temperature, followed by CD spectral acquisition. The melting temperature (Tm) was calculated from a thermal unfolding profile, generated by plotting the ellipticity change at 222 nm as a function of temperature. All CD spectra were averaged from three scans, followed by buffer correction. Each CD spectrum was reported as mean residue ellipticity (MRE).

#### Thermal denaturation analysis using differential scanning calorimetry (DSC)

Thermal unfolding of 0.5 mg/mL HAD-II enzyme was performed in 1 mM HEPES buffer (pH 8.0). PerkinElmer DSC 6000 differential scanning calorimeter (PerkinElmer, USA) was utilized to monitor the heat capacity of the enzyme over a temperature range of 20 to 80°C (heating rate: 1°C·min^−1^). Additionally, thermogram processing (peak identification and baseline correction) was carried out.

#### Cellular thermal shift assay (CETSA)

The thermal stability of purified HAD-II protein was evaluated using a cellular thermal shift assay (CETSA)-based thermal aggregation technique, followed by SDS-PAGE and BCA protein quantification analyses. The protein stock was aliquoted into ten microcentrifuge tubes (50 μL per tube) and incubated at different temperatures (25, 30, 35, 40, 45, 50, 55, 60, 70, and 80°C) for 10 min. Heat-treated samples were rapidly transferred to ice to terminate additional temperature-induced protein unfolding, followed by centrifugation at 15,000 ×g for 10 min to separate soluble protein fractions from thermally aggregated precipitates. A portion of the resulting supernatants was utilized for soluble protein quantification using the Pierce BCA protein assay kit, while the remaining fractions were heat-denatured and analyzed using SDS-PAGE. Following electrophoresis, gels were stained in a ProteOrange Protein Gel Stain 5000X (Lumiprobe, Maryland, USA) working solution prepared in 7.5% acetic acid and subsequently washed with dye-free 7.5% acetic acid before visualization.

##### Enzyme assay and defluorination efficiency

For the determination of defluorination activity, recombinant *E. coli* BL21(DE3) cells harboring the HAD-II-pET21a-LIC construct, obtained via transformation and subsequent overnight culture, were cultured in 50 ml of 1/10-strength Miller′s LB broth and incubated at 37°C until reaching an optical density (OD600) of 0.4–0.6 within 3–4 h. The culture was induced with IPTG and simultaneously spiked with 5 ppm PFOA. The reactors were then incubated at 40°C with continuous agitation for 24 h. In addition to the empty-vector *E. coli* BL21(DE3) control, cell-free media with PFOA were included to control for potential chemical defluorination and physical adsorption of PFOA to the biomass, respectively.

For purified HAD-II, in vitro assays were performed in a total volume of 2 ml of 50 mM HEPES buffer (pH 8.0) supplemented with 5 mM MgCl_2_ as the required cofactor and 5% (v/v) glycerol. The reaction began upon the addition of recombinant HAD-II (0.5 mg/mL) in the presence of 5 ppm PFOA, followed by incubation at 40°C under continuous agitation for 24 h. The enzyme-free control, consisting of buffer and substrate only, was incubated under identical conditions in parallel to correct background fluoride release.

For both whole-cell and purified enzyme assays, fluoride ion release was quantified using complementary ion-selective electrode (ISE) and ion chromatography (IC) methods. Regarding ISE, an ion-selective electrode system (Mettler Toledo SG78-FK2 SevenGo Duo Pro) with the sensitivity of approximately 1 × 10^−6^ mol/L was used to quantify fluoride ion content. For both bacterial cultures and purified enzyme reactions, aliquots were first centrifuged to remove cellular debris and enzyme/protein, respectively. Due to the low baseline concentration of fluoride in the samples, the clarified samples were mixed with TISAB III (instead of TISAB II) buffer at a 1:10 volume ratio to keep the fluoride concentrations safely above the electrode's lower detection limit while successfully stabilizing the analytical matrix before fluoride detection. To optimize measurement accuracy over the target experimental range, a linear calibration curve was constructed using a base-10 logarithmic transformation of standards from 0.02 to 5.00 ppm. Mean potential values (mV) and standard deviations of the calibration points, which are presented in **Table S2**, confirm Nernstian linearity (R^2^ > 0.99, slope ≈ −58.25 mV/decade). All readings were performed at room temperature to ensure electrode stability.

To further validate fluoride release, parallel quantification was conducted using a Dionex ICS-1500 Ion Chromatography (IC) system equipped with an AS50 autosampler and a high-sensitivity conductivity detector. Isocratic separation was performed on a Dionex IonPac AS18 analytical column at 30°C, using an 18 mM potassium hydroxide mobile phase at a constant flow rate of 1.0 mL/min and a 25 μL injection volume. Under the above-mentioned conditions, the diagnostic fluoride peak was consistently resolved at 3.0 ± 0.1 min. Peak assignments were confirmed by direct comparison with certified calibration standards, matching the manufacturer's reference chromatogram. A linear calibration curve (0.01–5.00 ppm) was constructed using certified standards. Mean peak areas and standard deviations of calibration points are detailed in **Table S3**, verifying system linearity (R^2^ > 0.99).

##### Biochemical analysis of HAD-II

###### Divalent cations

The HAD-II activity in response to divalent cations was examined by supplementing the reaction mixture with MgCl_2_ (5–30 mM) and individual metal salts (MnCl_2_, CaCl_2_, ZnCl_2_, and CoCl_2_ at 10 mM). To confirm metal dependency, 10 mM EDTA was added as a negative control instead of MgCl_2_ to chelate residual divalent ions.

###### Thermal behavior and stability of the enzyme

Based on the thermal stability profile obtained from CETSA analysis, temperatures between 35 and 45°C were selected to evaluate the thermal behavior and stability of the HAD-II enzyme. To assess temperature dependence, reaction mixtures were incubated within the above-mentioned temperature range at a pH of 8.0 for 24 h. In addition, thermal stability was determined by pre-incubating the enzyme for 4 h at temperatures between 35 and 45°C, followed by activity measurement upon addition of PFOA and incubation at 40°C and pH of 8.0 for 24 h. Enzyme-free blanks were included under identical conditions for all temperatures to correct for non-enzymatic fluoride release.

###### pH-dependent enzyme activity and stability

The pH effect on enzyme activity was evaluated by performing reactions in 50 mM buffers: HEPES (pH 7–8.5) and Tris-HCl (pH 8–9.0) at 40°C for 24 h. To evaluate pH stability, the enzyme was first kept on ice for 4 h under different pH conditions, followed by activity measurement at 40°C in enzyme buffer for 24 h. Blank controls without enzyme for each pH were used to correct background fluoride release signals.

###### Computational modeling and molecular dynamics simulations of HAD-II

Semi-flexible molecular docking was performed using AutoDock Vina^54,55^ with the HAD-II crystal structure (predicted using the AlphaFold server). The atomistic structure was analyzed in ChimeraX, and the PFAS ligand was prepared in PyMOL^56^. Docking was centered at coordinates (x = 3.4, y = 0.2, z = −1.9) with grid dimensions of (x = 15, y = 15, z = 10). Each run was repeated five times, and the pose with the lowest binding energy was selected and visualized in ChimeraX^57^.

Molecular dynamics (MD) simulations were carried out in GROMACS 2025.2 using the CHARMM36 force field. Systems were prepared in VMD, equilibrated, and simulated for 500 ns with a 2 fs time step at 325 K. The average structure was obtained using the gmx cluster tool for further analysis. For structural homology analysis, HAD-II fold conservation is confirmed by Boltz-2 (v2.0) with multiple sequence alignment (MSA) generation via the ColabFold MSA server. Structural quality was assessed using the predicted TM-score (pTM) and per-residue pLDDT confidence metrics. Structural homology searches were performed using Foldseek (v8.ef4e960) with TM-align scoring against the RCSB PDB database, retaining up to 50 hits per query at an e-value threshold of 0.01. Pairwise structural similarity between the two predicted models was computed using Foldseek TM-align. TM-scores > 0.5 indicate the same overall fold, and TM-scores > 0.7 indicate the same structural superfamily.

###### Phylogenetic analysis of HAD-II Dehalogenase sequences

Protein sequences were clustered at 80% identity using CD-HIT (v4.8.1) to obtain 45 representative sequences. Multiple sequence alignment was performed with MUSCLE (v5.3) and trimmed with trimAl (automated mode), yielding 238 alignment columns. Maximum likelihood phylogenetic inference was performed with IQ-TREE2 (v3.1.1) using ModelFinder (BIC criterion; best model: Q. PFAM+G4) and 1000 ultrafast bootstrap replicates (UFBoot). The tree was midpoint rooted. To test for long-branch attraction (LBA), three additional IQ-TREE2 analyses were performed: (i) the full alignment under LG + F+G4 (AIC-selected model), (ii) the full alignment under Q.PFAM+R4 (free-rate model, more LBA-resistant), and (iii) a reduced alignment excluding Xanthobacter sp. (the longest branch). The placement of the study enzyme was consistent across all four analyses, ruling out LBA as an explanation for the observed topology. Pairwise percent identity between the study enzyme and each of the 57 homologs was computed from the trimmed multiple sequence alignment using a gap-aware formula: identity (%) = (number of identical aligned positions excluding columns where either sequence has a gap) / (number of aligned positions without gaps in either sequence) × 100. Amino acid similarity was additionally assessed using the BLOSUM62 substitution matrix. The multiple sequence alignment (MSA) figure was generated for 5 representative sequences ordered by their tip positions in the maximum-likelihood tree. The coloring schemes were applied to the physicochemical class in accordance with the ClustalX convention.

###### Direct-infusion MS analysis of PFOA defluorination products

Following enzymatic treatment, reaction mixtures were clarified by centrifugation at 12,000 ×g to remove enzymes/proteins and insoluble materials. The supernatant was subjected to weak anion-exchange solid-phase extraction (WAX-SPE) for cleanup, desalting, and solvent exchange from aqueous buffer to methanol. The SPE eluate was also diluted with methanol to obtain a final PFOA concentration of 0.5 ppm from the original 5 ppm PFOA reaction sample to prevent detector saturation during qualitative analysis. The same SPE extraction and dilution steps were applied to the untreated PFOA sample to serve as the analytical control. Mass spectrometric analysis was performed through direct infusion in negative-ion mode using an LTQ XL^™^ Linear Ion Trap Mass Spectrometer (Thermo Fisher Scientific, USA) over the range of m/z 100–450 to track residual PFOA and possible defluorination product ions. The appearance or increase of new ions after enzymatic treatment was evaluated by comparison with the untreated PFOA control.

###### Data processing and analysis

All datasets were analyzed using Origin (OriginLab, USA), and data are presented as mean ± standard deviation (SD).

## Supplementary Material

Supplementary Files

This is a list of supplementary files associated with this preprint. Click to download.

- 3.SupplementaryInformationV8.docx
- 4.GraphicalAbstract.pdf

## Data Availability

All experimental and computational data supporting the findings of this study are available at Zenodo under 10.5281/zenodo.20517596. All other additional information related to the article is provided in the Supplementary Information.
